# New tricks for an old dog: opportunities for better tuberculosis control

**DOI:** 10.1002/jia2.26081

**Published:** 2023-03-23

**Authors:** Digby F. Warner, Robin Wood

**Affiliations:** ^1^ Molecular Mycobacteriology Research Unit & DSI/NRF Centre of Excellence for Biomedical TB Research Department of Pathology Faculty of Health Sciences University of Cape Town Cape Town South Africa; ^2^ Institute of Infectious Disease and Molecular Medicine Faculty of Health Sciences University of Cape Town Cape Town South Africa; ^3^ Wellcome Centre for Infectious Diseases Research in Africa Faculty of Health Sciences University of Cape Town Cape Town South Africa; ^4^ Desmond Tutu Health Foundation Faculty of Health Sciences University of Cape Town Cape Town South Africa

1

Tuberculosis (TB) remains an enigmatic disease. Consistently among the leading causes of annual global mortality, its impact is greatest in a limited number of high‐burden countries, many of which are marked by poverty, stark internal inequalities, societal instability and, since the late 20th century, high HIV prevalence. An ancient disease which has plagued humanity for millennia, TB often defies definitive diagnosis, especially in those most at risk, such as children and people living with HIV, where presentation can be complicated owing to immature or deficient immune function. Declared a global emergency in 1993, TB struggles perennially to secure a position in the popular consciousness (in contrast to COVID‐19), and consistently fails to attract the funds necessary to drive preventative and therapeutic interventions, as well as the research required to develop innovative new programmes [1].

Programmatic control and clinical management of TB require a deeper understanding of the complex interaction between the causative agent, *Mycobacterium tuberculosis*, and its obligate human host. TB control remains heavily dependent on the treatment of active disease, with the standard 6‐month multidrug combination therapy posing a host of challenges for health systems, in conjunction with poor patient adherence. Key recent developments include innovations in composition and duration of combination therapies for both drug‐susceptible and drug‐resistant disease [2], and the potential to accelerate new regimen development through adaptive clinical trial design [3]. In addition, through the efforts of pioneering partnerships, such as the Tuberculosis Drug Accelerator [4], the pre‐clinical pipeline for new TB drugs is stronger than ever [5], harnessing advanced technologies to address knowledge gaps, such as the distributions of anti‐TB drugs into diverse anatomical compartments [6], and the optimal design of new combinations to reduce treatment durations and limit the development of drug resistance [7].

Effective therapy depends on knowledge of who is ill, and the development of improved point‐of‐care TB diagnostics remains a priority. Recent advances include expanding the GeneXpert molecular assay to detect multi‐drug resistance [8], the use of whole‐genome sequencing direct from clinical samples [9] and biomarker‐based approaches to diagnose active disease [10]. Artificial‐intelligence‐based computer‐aided detection systems for rapid digital X‐ray diagnosis are also showing promise [11]. Novel technologies—not yet ready for widespread implementation—include the use of face‐mask sampling [12] and the collection of breath aerosols [13]. The addition of newer, non‐sputum‐based diagnostics is critical to better define the TB disease state.

Advancing new vaccines, to replace Bacille Calmette‐Guérin for the prevention of infection or to prevent progression to disease, is essential to make realistic progress towards global TB control, and a comprehensive roadmap outlines how this might be accelerated, based on recent experience of multiple ambitious, high‐profile endeavours [14]. Like drug development, these initiatives continue to benefit greatly from more complex models of *M. tuberculosis* infection and TB disease, such as those using non‐human primates [15].

And what of the immediate future? Regaining ground lost to COVID‐19 is critical but will not be easily achieved. There are, however, some encouraging signs suggesting lessons from that pandemic can be usefully applied to TB. For example, it was not long into the COVID‐19 pandemic that the concept of subclinical SARS‐CoV‐2 infection was assimilated into mainstream thinking. It seems strange, therefore, that an analogous appreciation of the potential for subclinical—even asymptomatic—*M. tuberculosis* infection and transmission as major drivers of TB prevalence in high‐burden settings has been so reluctantly adopted. There are some major uncertainties: what is the population‐attributable proportion of transmission from the subclinical population, how can active case finding be sustainably implemented and should all subclinical *M. tuberculosis* infections be treated and with which regimens? In some ways, the hesitation to acknowledge subclinical infections as potentially major sources of TB transmission events might stem partially from history. Robert Koch developed his paradigmatic postulates to convince a then‐sceptical audience that *M. tuberculosis* was the etiological agent of TB. A spurious corollary of causation, though, is that lack of overt disease signifies absent organisms—that is, subclinical infection renders negligible risk of transmission. This reasoning has unfortunately informed many approaches to TB control.

Evidence supporting the contribution of subclinical disease to high TB prevalence rates appears incontrovertible, and, notably, fits with a large historical literature describing the presence of TB lesions and/or infectious *M. tuberculosis* organisms in undiagnosed individuals after death from other causes [16]. For example, a recent National TB Prevalence survey in South Africa [17] reported that the prevalence‐to‐notification ratio exceeded 1 across all age groups sampled, approaching 3 in two cohorts (15–24 years; ≥65 years). The observation that more than half of all bacteriologically confirmed pulmonary TB was asymptomatic indicates chronic under‐diagnosis in this high‐burden setting—the consequence being continued transmission from undetected *M. tuberculosis* infections.

The importance of subclinical infections in TB transmission is reinforced by studies utilizing face‐mask sampling [12] and aerosol capture [13] technologies to demonstrate, respectively, the capacity for cough‐independent *M. tuberculosis* release and the potential contribution of normal breathing to bacillary aerosolization. These and related results have added impetus to the growing recognition of subclinical *M. tuberculosis* infections as the next major frontier in TB control [18]. The implications are not trivial, however, and raise several tough questions around the development and deployment of vaccines in high‐prevalence settings, how diagnostic thresholds will be set as detection technologies achieve increased sensitivities and how best to use anti‐TB chemotherapy. Nevertheless, the benefits accruing from a much more nuanced understanding of the drivers of disease and the risks of transmission must surely outweigh any concerns about reframing the TB control framework.

The potential for subclinical infection reasserts the axiom that *M. tuberculosis* is necessary but not sufficient for TB. Importantly, by relaxing the tight causal link between the bacterium and the disease, it re‐focuses attention on the host as an essential variable impacting the outcome of infection. The surpassing need now is to understand the biological factors which lead to TB susceptibility, morbidity and mortality, and to confront the social inequities which continue to condemn large numbers of people in high‐burden regions to elevated risks of disease progression. We have some of the tools, and COVID‐19 has demonstrated that a huge impact is possible with concentrated, concerted effort.

## COMPETING INTERESTS

The authors have no competing interests to declare.

## AUTHORS’ CONTRIBUTIONS

DFW and RW equally contributed to the conceptualization, drafting and writing of the manuscript, and approved the final version.

## FUNDING

Work in the Warner and Wood laboratories on tuberculosis transmission is supported by the South African Medical Research Council as well as the National Institute of Allergy and Infectious Diseases of the US National Institutes of Health under award number R01AI147347 and through the Myco3V Tuberculosis Research Unit (U19AI162584).
